# The Transition to Caregiver in Advanced Alzheimer’s Disease: From Emotional Connection to Care Responsibility—A Grounded Theory Approach

**DOI:** 10.3390/nursrep15080284

**Published:** 2025-08-04

**Authors:** Federica Dellafiore, Orejeta Diamanti, Luca Guardamagna, Gloria Modena, Pierpaolo Servi, Donato Antonio Rotondo, Tiziana Nania, Andreina Saba, Giovanna Artioli

**Affiliations:** 1 Department of Life Health Sciences and Health Professions, Link Campus University, 00165 Rome, Italy; 2 Healthcare Professions, Veneto Institute of Oncology IOV—IRCCS, 35128 Padua, Italy; orejeta.diamanti@iov.veneto.it; 3 Department of Orthopedics and Traumatology, Istituti Clinici di Pavia e Vigevano S.p.A., 27100 Pavia, Italy; luca.guardamagna@grupposandonato.it; 4 Welfare Services Area, Irccs Humanitas Research Hospital, 20089 Rozzano, Italy; maria.modena@humanitas.it; 5 Division of Neonatal Intensive Care Unit, Foundation IRCCS San Matteo Hospital, 27100 Pavia, Italy; pierpaoloservi@gmail.com; 6 Emergency Medical Service 118, Brindisi Local Health Authority, 72100 Brindisi, Italy; donato.rotondo@gmail.com; 7 Training Office, IRCCS Policlinico San Donato, 20097 Milano, Italy; tiziana.nania@grupposandonato.it; 8 Department of Primary Care Ausl Irccs Reggio Emilia, 42122 Reggio Emilia, Italy; andreina.saba@ausl.re.it (A.S.); giovanna.artioli@unipr.it (G.A.)

**Keywords:** caregivers, lived experience, grounded theory, Alzheimer’s disease

## Abstract

**Background:** The progression of Alzheimer’s Disease (AD) deeply affects not only the diagnosed person but also their close relatives, who are often called to take on the role of informal caregivers. This transition is frequently unplanned and emotionally complex, yet poorly understood in its deeper processual dimensions. This study aims to explore and theorize the transition experienced by a family member becoming the primary informal caregiver for a person with advanced AD. **Methods:** A qualitative study based on the Constructivist Grounded Theory according to Charmaz’s approach (2006) was conducted. In-depth interviews were carried out with 10 participants who had become informal caregivers for a loved one with advanced AD. Data were analyzed using initial coding, focused coding, the constant comparative method, and theoretical coding. **Results:** Ten caregivers (mean age 39 years, range 35–54; nine females) of patients with advanced AD participated in the study. The analysis revealed a complex, emotionally intense caregiving experience marked by sacrifice, feelings of powerlessness, identity loss, and the necessity of sharing caregiving responsibilities. A core category emerged: A Silent and Certain Willingness to Care, representing the caregivers’ deep, often unconscious commitment to prioritize the care of their loved ones above their own needs. Four interconnected phases characterized the caregiving process: (1) The Changing Daily Life—involving significant sacrifices in personal and social life; (2) Feeling Powerless—confronting the inevitable decline without means to alter the course; (3) Losing Oneself—experiencing physical and psychological exhaustion and a sense of identity loss; and (4) Sharing with Others—seeking external support to sustain caregiving. These findings highlight the evolving nature of becoming a caregiver and the enduring dedication that sustains this role despite the challenges. **Conclusions:** The progression of AD deeply transforms the lives of caregivers, who become co-sufferers and active participants in the disease’s management. The results underscore the urgency of designing integrative care strategies—including psychological, social, and potentially technological support—that can enhance both patient outcomes and caregiver resilience. Grounded in real-world experiences, this study contributes to the broader neurodegeneration discourse by emphasizing caregiving as a critical factor in long-term disease management and therapeutic success.

## 1. Background

Alzheimer’s disease (AD) represents an increasingly pressing public health issue [1]. It currently affects over 50 million people worldwide, and this number is projected to triple by 2050 due to global population aging [1,2]. The risk of developing AD rises significantly with age, and by 2040, it is estimated that approximately 81 million individuals over the age of 60 will be living with the disease globally [3]. In Italy, around one million people are currently living with dementia, with AD accounting for an estimated 50–60% of cases, in addition to many others experiencing cognitive decline [4]. To date, no pharmacological treatment has been proven to halt or reverse the progression of AD; available therapies are limited to managing symptoms [5].

As a result, AD patients face a chronic, irreversible, and often profoundly distressing condition that also deeply affects family systems and caregivers [4]. This condition inevitably and profoundly impacts the patient’s surrounding environment, including family routines, emotional relationships, and, above all, the informal caregiver [3]. The informal caregiver is the person who provides care, support, and assistance to loved ones with AD—without formal training or compensation—helping them in daily activities and medical needs. Specifically, caregivers can be classified as formal—professional healthcare providers—and informal, who are usually family members or close friends [6]. The informal caregiver carries the greatest burden, often assuming most responsibility for managing the disease and its effects on the patient’s life. This role is demanding and emotionally taxing, highlighting the critical importance of support for caregivers in the overall care of individuals with AD [7].

In the Italian context, informal caregiving remains deeply rooted in cultural values of filial responsibility, intergenerational solidarity, and moral duty. Among EU countries, Italy is one of the few without a national law that legally recognizes and supports informal caregivers. More than 7 million Italians—approximately 15% of the population—are involved in informal caregiving for family members [8]. Women, particularly daughters and spouses, are predominantly the ones who become caregivers, which reflects traditional gendered expectations in family caregiving roles. Most caregivers are over the age of 50, and one in five is over 60. One recent estimate indicates that 63% to 75% of caregivers are women, mostly aged 45–64 [9]. For nearly half of them, the weekly time commitment to caregiving ranges between 10 and 20 h, while one-quarter dedicate more than 20 h weekly [8]. Despite this widespread phenomenon, dedicated policies and structured support services are still limited. As far as the Italian contribution to research is concerned, there are still few groups working in this area. Notably, in 2015, the UP-TECH research group was born, with the aim of supporting caregivers of AD patients in Italy [10].

Given the progressive cognitive, functional, behavioral, and psychological decline associated with AD, families are increasingly taking on a central role in care provision, with the burden rising significantly as the disease progresses [7]. Specifically, despite the involvement of formal caregivers (e.g., paid care workers), the primary responsibility typically falls to informal family caregivers [11]. Caring for a patient with advanced AD can profoundly impact multiple aspects of a caregiver’s life. As the disease progresses, caregivers frequently face considerable emotional distress, including heightened levels of anxiety and depression, along with increased caregiver burden, a decline in quality of life, financial strain, and insufficient support [12]. Moreover, caregivers must continuously adapt to the unpredictable course of the disease, managing and making sense of their loved one’s evolving symptoms on a daily basis [13,14].

Comparative evidence suggests that the experience of caregiving can differ significantly according to the type of illness. For instance, caregivers for patients with late-stage cancer often experience a time-limited but intense burden focused on symptom management and end-of-life planning [15], while caregivers for relatives with severe mental disorders (such as schizophrenia) face ongoing challenges related to behavioral unpredictability, stigma, and the need for long-term social support [16]. In all these contexts, family caregivers—most commonly women and close relatives—must develop illness-specific skills and coping strategies to manage their loved one’s needs effectively. This highlights that while the moral willingness to care is a common thread, the practical and emotional demands vary across different conditions.

Despite the disruptions to daily life, many caregivers feel a strong sense of responsibility and moral obligation toward their loved ones [17]. As AD follows an unpredictable trajectory, caregiving roles evolve significantly over time. Family dynamics often shift, and caregivers may sacrifice personal aspects of their lives to maintain balance in care [18]. Gender and the nature of the relationship with the patient further shape caregiving experiences, which typically unfold across several stages—from initial symptom monitoring to long-term planning and end-of-life care [13,19]. Caregiving becomes deeply embedded in daily routines, involving tasks like meal preparation, medication management, and financial oversight [19]. A grounded theory study identified five caregiving phases—each linked to distinct emotional, informational, and practical needs—offering a useful framework to understand how caregiver demands shift over time [19]. Additionally, literature showed that older spousal caregivers (OSCs) face unique challenges, often shaped by cultural context. A study in Iran, for example, identified “getting sincere and efficient support” as central to their experience, yet also highlighted significant barriers to both formal and informal support [20].

While previous research—including influential contributions such as Montgomery, Rowe, and Kosloski (2007) [21]—has explored the shifting responsibilities and identity of family caregivers for frail or disabled older adults more generally, there remains a notable gap in understanding the processual transition of family members becoming primary informal caregivers specifically for a person with advanced AD. Much of the existing literature has focused on general caregiving stages, practical responsibilities, or support needs, but has often overlooked the deeper transformative process through which individuals renegotiate their identity, relational boundaries, and daily life in the face of progressive cognitive decline. This process is also embedded within a unique cultural context in Italy, where family solidarity often replaces formal services, making informal caregiving a critical yet under-researched phenomenon. The transition from an emotional bond rooted in kinship to a caregiving role shaped by routine, responsibility, and increasing dependency is especially complex in advanced AD, yet remains underexplored in its relational and experiential dimensions.

This study seeks to address this gap by exploring and theorizing the lived transition from family members to informal caregivers within the context of advanced AD, using a Constructivist Grounded Theory approach. By focusing on how individuals navigate the emotional, relational, and practical shifts involved in assuming a caregiving role, this research aims to deepen our understanding of this critical process. Ultimately, the goal is to inform the development of culturally sensitive, stage-specific interventions that acknowledge and support the evolving identity and needs of informal caregivers facing the challenges of AD.

## 2. Methods

### 2.1. Study Design

A qualitative study was conducted using a constructivist grounded theory (CGT) approach [22] to explore the process through which a family member becomes a caregiver for a loved one living with advanced AD. The central research question guiding the study was: “*What are the lived experiences of a family member transitioning into the caregiver role for someone with advanced AD?*” This question required an in-depth exploration of the complex biopsychosocial and emotional processes involved in caregiving within a context of progressive cognitive decline.

CGT is particularly well-suited to investigating social processes and human behavior, as it posits that reality is not objective but co-constructed through interactions and shared meanings [22,23]. This epistemological stance informed the selection of CGT as the most appropriate methodological framework for the study. CGT is especially valuable when existing knowledge is limited, enabling researchers to construct new understandings of psychosocial phenomena. CGT offers a systematic yet flexible approach to qualitative research, allowing for the simultaneous collection and analysis of data. This iterative process supports the development of theoretical insights that emerge directly from empirical evidence rather than from preconceived assumptions [22,24].

In the constructivist approach, researchers do not remain detached observers but assume an active and participatory role alongside study participants. They engage collaboratively throughout the research process, contributing their perspectives and experiences while simultaneously acknowledging and valuing those of the participants. This interactive engagement allows for the co-construction of knowledge and shared meaning, emphasizing that understanding emerges dynamically within the specific social and cultural context of the study [25]. To enhance the rigor of the analysis, multiple strategies were employed to ensure the credibility, dependability, and confirmability of the findings. These included constant comparative analysis, detailed memo-writing, independent coding by multiple researchers with subsequent discussion to resolve discrepancies, and member checking with participants to verify the accuracy and resonance of emerging categories. Such measures reflect best practices in qualitative research and grounded theory to establish methodological trustworthiness in lieu of traditional quantitative notions of reliability and validity. To ensure methodological rigor, the study followed the 32-item Consolidated Criteria for Reporting Qualitative Research (COREQ) checklist [26].

### 2.2. Sampling

We employed both initial and theoretical sampling, consistent with the principles of Grounded Theory [27]. Initial sampling was purposive and guided by the study’s aims, involving the deliberate selection of participants with direct experience and knowledge of the phenomenon under investigation [25]. In our case, the study population consisted of family members of AD patients who had agreed to assume the role of caregiver and provide care for their relatives. The following inclusion criteria were also met: (a) having a family member diagnosed with AD; (b) living with the AD patient; (c) non-cohabiting individuals who fulfilled all other criteria and demonstrated primary caregiving responsibilities; (d) having been engaged in caregiving activities for at least 12 months, to ensure participants have sufficiently experienced the caregiving role and can provide in-depth perspectives; (e) being available to participate in the study and willing to share their personal history, perspectives, and experiences; and (f) having a good command of the Italian language.

Recruitment took place in local community settings, including AD’s family support associations and outpatient memory clinics in [specify region or city, e.g., Northern Italy]. Potential participants were identified with the support of healthcare professionals and caregiver networks. A researcher experienced in Constructivist Grounded Theory methodology provided detailed information about the study and contacted eligible caregivers by email or phone to request their availability. Face-to-face interviews were then arranged at locations convenient for the participants, such as their homes or private rooms in community centers. No financial reimbursement was offered for their time; however, participants were fully informed about the study aims, the voluntary nature of participation, and the value of their contribution to improving support for family caregivers. Participants were initially recruited through purposive sampling, and subsequently, theoretical sampling was employed to guide data collection [28]. Specifically, theoretical sampling was applied in the later stages of the research to support constant comparison and achieve saturation of emerging categories during focused coding [25,27]. This approach allowed us to select participants based on theoretical insights and evolving conceptual needs.

### 2.3. Data Collection

Semi-structured interviews were conducted between September and December 2024 by a researcher with extensive expertise in qualitative interviewing, ensuring consistency in data collection and adherence to ethical standards, particularly regarding confidentiality. Specifically, recognizing the demands of conducting rigorous grounded theory interviews, all members of the research team received specific training in grounded theory methodology and qualitative interviewing techniques, ensuring high methodological quality and responsiveness to the evolving nature of the data collection process.

A total of ten participants were recruited through purposive sampling based on criteria relevant to the research aim, ensuring diversity in caregiving experiences. The final sample included ten informal caregivers of persons diagnosed with advanced AD. Participants ranged in age from 35 to 54 years (mean: 39 years). The majority were female (n = 9), and most were either spouses (n = 1) or adult children (n = 9) of the care recipient. Six caregivers were co-residing with the patient, while four lived elsewhere: despite not cohabiting, these four caregivers were included as they provided primary informal care comparable to co-residing participants. The duration of their caregiving experience varied from 3 to 11 years (mean: 8.3 years). To capture a range of perspectives, participants with varying socioeconomic backgrounds, levels of formal support, and living arrangements were included. Recruitment continued until theoretical saturation was achieved, consistent with grounded theory methodology. All participants provided informed consent prior to taking part in the study.

The care recipients were aged between 72 and 88 years. The duration since diagnosis of AD ranged from 2 to 10 years, and all patients were perceived by caregivers as being in an advanced stage of AD. Common complications reported included mobility impairments, incontinence, and behavioral disturbances, which added complexity to the caregiving role. Although detailed clinical data were not systematically collected for all patients, these characteristics emerged from caregiver accounts and helped contextualize the caregiving experience.

The interview guide was developed prior to data collection and included semi-open questions designed to explore the caregiving experience in depth (Appendix A). An initial open-ended question—such as “*Can you tell me about your experience as a caregiver for your loved one with Alzheimer’s disease?*”—was used to foster an open, non-judgmental atmosphere, followed by questions specifically addressing the caregiving role as experienced by family members [24]. The design of the interview guide was informed by Charmaz’s (2014) [25] intensive interviewing approach, which emphasizes flexibility and responsiveness during the interview process. This allowed conversations to unfold naturally, with the interviewer introducing prompts to encourage narrative depth and the emergence of participants’ lived meanings. The initial questions were aligned with the study’s central aim of exploring the caregiving experience in the context of AD, seeking to uncover challenges related to diagnosis, care dynamics, and personal adaptation [14,24].

As the study progressed, theoretical sampling guided adaptations to the interview process. A modified version of the original guide was used to include more focused questions derived from the emerging analytical categories. This iterative approach allowed the interview content to evolve in response to developing theoretical insights, thereby supporting the grounded theory methodology. To capture the complexity and nuance of participants’ experiences, six individuals were interviewed twice, allowing for greater depth and clarity in the data.

### 2.4. Memoing, Reflexivity, and Rigor

Throughout the research process, memo-writing played a central role in shaping and deepening data analysis. This practice supported an ongoing dialogue among the researchers, enabling the refinement of categories and the emergence of new conceptual insights in line with Charmaz’s recommendations (2014) [25]. As highlighted by Birks et al. (2008), memoing fosters immersion in the data, enhancing researchers’ sensitivity to implicit meanings [29]. Moreover, memos served not only as analytical tools but also as a means of engaging in reflective critique, allowing the team to examine potential preconceptions and biases [30]. In this study, memo-writing was particularly valuable in reinforcing collective awareness of the evolving theoretical framework [25].

To ensure methodological rigor and credibility [31,32,33], the research process began with a deliberate alignment between the study’s objectives and its constructivist grounded theory design. Under the mentorship of a qualitative research expert, the team adopted a collaborative approach to analysis, working together to construct and interpret emerging meanings. Consistent with the constructivist perspective, the use of shared memos supported the development of theoretical ideas while maintaining a balance between the researchers’ interpretative lens and participants’ lived experiences.

A coherent strategy was also maintained during data collection and analysis phases, with regular debriefings and post-interview discussions to enhance reflexivity and analytical clarity. Although participants did not take part in coding or interpretation, a final seminar was held to present preliminary findings. This session invited feedback from participating healthcare professionals and patients, allowing them to reflect on and comment upon the emerging theoretical model.

### 2.5. Data Analysis

Interview recordings were transcribed verbatim by members of the research team and subsequently verified by the interviewer to ensure accuracy. Data analysis was conducted concurrently with data generation, following the principles of Constructivist Grounded Theory. We applied open, focused, and theoretical coding. Initially, open coding was performed on the first six interviews by two researchers using an inductive and constant comparative method. This phase was then extended to include three additional interviews. Segments of the transcripts were labeled with codes, and the researchers collaboratively agreed on a unified code list, which was subsequently reviewed by L.G. and discussed with the entire team.

Focused coding involved grouping conceptual codes and organizing them into provisional categories (n = 16), a task carried out by researchers. These categories aimed to reflect the emotions and perspectives of participants and were arranged chronologically to represent the caregiving journey. This temporal framework served as a foundation for deeper theoretical exploration.

**Figure 1:**
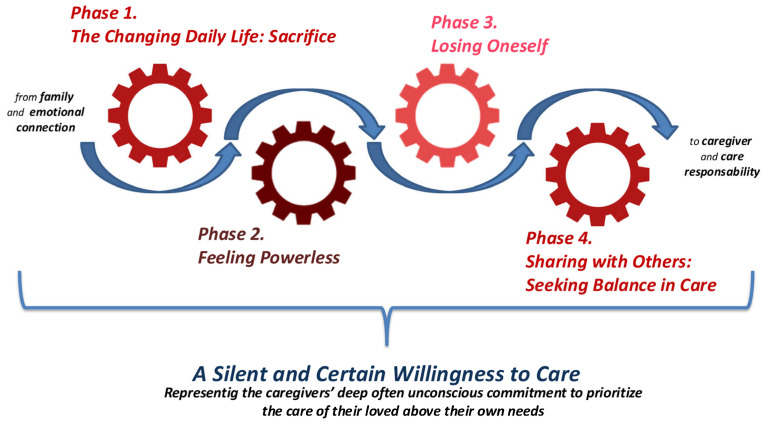
Conceptual Map of Categories Refined Through Theoretical Sampling and Coding.

As the analysis progressed, provisional categories were continuously compared against the full dataset. Theoretical coding and sampling allowed us to refine the categories and clarify their interrelationships, aligning them both chronologically and conceptually (Figure 1). Data collection concluded when theoretical saturation was achieved, and the final analysis produced four main categories and nine related sub-categories.

Clinical and contextual information regarding the care recipients was collected through caregiver interviews, during which participants were invited to describe relevant aspects of the patient’s health status, disease progression, and care needs. These qualitative data provided insight into the clinical complexity and caregiving challenges, although no formal clinical assessments or medical records were accessed as part of this study. This approach aligns with the constructivist grounded theory methodology, which prioritizes participants’ subjective experiences and perspectives to understand the phenomenon under investigation.

## 3. Results

The sixteen interviews conducted revealed emotionally intense, often contradictory narratives that reflect the complex, evolving, and processual nature of becoming a caregiver for a person with advanced AD.

Analysis revealed a dynamic, transformative, and continuous process that is neither linear nor predictable. It is characterized by overlapping, recursive, and interwoven phases, demonstrating that caregiving is not a static condition, but an ongoing adaptation shaped by multiple factors, including the progression of the disease, the nature of the relationship with the care recipient, and the available support systems. The process is framed by a core category and unfolds through four major phases, as illustrated in Figure 1, which visually represents the non-linear and fluid trajectory of this caregiving journey.

### 3.1. Core Category: A Silent and Certain Willingness to Care

The core category emerging from the data reflects a profound, often unconscious, yet unwavering willingness to care for loved ones affected by advanced AD. Caregivers consistently demonstrated a quiet commitment—an intrinsic readiness to prioritize the patient’s needs over their own, sacrificing personal time, habits, and priorities without conscious deliberation. “*You can’t say no. There’s no choice. She’s your mother, that’s it.*” (P4); “*I never stopped to think ‘can I do this or not?’. It had to be done.*” (P6); “*What you do is not a job. It’s your mother*” (P2). This willingness is not described as a conscious choice or negotiated decision, but as an inherent and almost unquestionable sense of duty. As one participant explained: “*I gave up a lot of myself… that’s perhaps the most dramatic thing that happened… I can’t just go to the gym or rest more because I have to help her in the morning*” (P6). Another caregiver highlighted this total dedication, stating, “*I left my job intentionally to follow her… I decided to dedicate myself completely to her*” (P4). This quiet yet resolute commitment to caregiving permeates every aspect of daily life and shapes the ensuing phases of adaptation and coping “*There’s no point where you say, ‘now I become a caregiver’. It just happens to you.*” (P8) “*Everything fell on me, everything… I didn’t know where to turn*” (P4). Caregivers described profound changes to their routines and social lives: “*In those worst years, I no longer had a life… at night I was with my mother, during the day I worked, then came home… I didn’t see anyone anymore… and I didn’t even want to*” (P3). This continuous sacrifice underscores the certainty with which caregivers embrace their role, as if caregiving were the only possible path—a silent pact rooted deeply in their relationship with the patient.

### 3.2. Phase 1. The Changing Daily Life: Sacrifice

Caregivers reported feeling isolated and described not only a loss of social connections, but also a reduced desire to engage socially due to the physical and emotional toll of caregiving. Caregiving evolved from an act of love into an all-consuming experience that reshaped both personal and family life. One participant shared: “*In those worst years, I no longer had a life because at night I was with my mother, during the day I worked, then came home… I didn’t see anyone anymore… I didn’t even want to*” (P3). Another described the renunciation of personal activities: “*I gave up a lot of myself… that’s perhaps the most dramatic thing that happened… I can’t just go to the gym or rest more because I have to help her in the morning*” (P6). The new daily routine imposed severe limitations: “*The gym, parents’ groups, even just having coffee after lunch! It’s impossible to go out; you always have to be ready*” (P8). Social relationships suffered severely: “*You lose friendships because, well… who approaches you? No one! The first time you say no to a pizza, the second time they start to frown, the third time… it’s automatic*” (P9). For some, professional life was affected: “*No, I deliberately left my job to follow her… I worked as a sales assistant, and I quit when I discovered the illness. I decided to dedicate myself fully to her*” (P4).

### 3.3. Phase 2. Feeling Powerless

As the disease progressed, caregivers experienced a deep sense of helplessness, pain, and frustration. The awareness that the illness cannot be cured or stopped brought profound suffering.

Many described the anguish of watching their loved ones deteriorate: “You see someone aging fifty years in thirteen years… and you can do nothing… You try to help, but you can’t… when she tries to have conversations… The worst thing is seeing her struggle, but not being able to help because you don’t understand, and you can’t do anything” (P3). This sense of powerlessness intensified during moments of acute distress: “I felt powerless every time I couldn’t soothe my mother during nervous attacks… and when she had epileptic seizures, I could only watch without doing anything… The hardest part is witnessing the complete destruction of the human being” (P8). The emotional devastation was profound: “She used to call every Sunday… and seeing her disappear devastated me even more” (P6). This powerlessness did not stem from inaction but from the lack of control over the other person’s fate, and it was often accompanied by guilt or a sense of inadequacy: “You feel powerless, even when you give everything” (P9).

### 3.4. Phase 3. Losing Oneself

The caregiving journey had a deep impact on caregivers’ physical and mental well-being, leading to exhaustion and identity loss. One caregiver shared: “*I am physically and psychologically exhausted… I experienced a sharp drop in energy and vitality… I have great difficulty even with the simplest daily tasks*” (P4). Another noted strained family relationships: “*I have my own family nearby… but I spend more time at her house than mine… Sometimes my daughter accuses me of loving my grandmother more than her… Maybe if I had followed her schoolwork better, she would have had fewer difficulties, but you can’t know*” (P8). In some cases, the emotional burden led to panic attacks: “*At a certain point, I had panic attacks… I lose control because my brain reaches a limit it can’t handle anymore*” (P6). This phase is often silent, lacking external recognition, yet it marks a profound fracture in personal identity: “*I didn’t feel like myself anymore. I didn’t recognize myself.*” (P7).

### 3.5. Phase 4. Sharing with Others: Seeking Balance in Care

Due to the intense physical and emotional strain, many participants acknowledged the need to share caregiving responsibilities. Some sought external help to manage daily tasks and regain personal time. The arrival of external support (such as a caregiver or assistant) represents a turning point: it lightens the burden and opens the door to self-recovery: “*Four months ago we hired a caregiver… now Elena is away until September… She is a good person and very attached… Now there’s someone from 8 a.m. all morning… and when Elena returns, maybe she’ll work full time… We have a good relationship with Elena now*” (P7). Another caregiver highlighted the importance of assistance during work hours: “*I obviously needed someone to look after my mother while I was at work because my job isn’t easy… We hired a caregiver for 6–7 h during the time I wasn’t there*” (P9). This support helped alleviate personal sacrifices: “*Since the caregiver arrived, I’ve had fewer renunciations… I always tell people in this situation, ‘Even if you don’t eat, get a caregiver.’ For me, it was like starting to live again*” (P3). The shift from ‘doing everything alone’ to ‘sharing care’ is delicate but essential to stop the erosion of identity and reclaim personal space.

## 4. Discussion

This study aimed to explore the process by which family members transition into the role of informal caregivers for individuals living with advanced AD. Utilizing Charmaz’s Constructivist Grounded Theory methodology, we captured the nuanced and evolving experiences of caregivers as they navigated the emotional, relational, and practical challenges of assuming this demanding role. Our findings position caregiving not as a fixed status but as an ongoing, negotiated, and relational process that continuously redefines both the caregiver’s identity and their everyday life. The resulting grounded theory sheds light on a complex trajectory shaped by emotional bonds, role negotiation, and moral commitment. Unlike previous research that tends to focus on the burden or stress of caregiving, this study brings to the forefront the existential and ethical dimensions of the caregiving experience, particularly as it relates to the transformative and processual nature of identity reconstruction and the intimate redefinition of familial relationships. The innovative value of this study lies in its focus on the *process* of becoming a caregiver—not merely the outcomes or impacts—and in its articulation of the caregiving journey as an evolving moral and emotional endeavor. These findings offer a deeper, more humanized understanding of caregiving in the context of neurodegenerative disease, with implications for both clinical practice and policy development.

The transformation experienced in becoming a caregiver is an internal, evolving process that extends beyond the mere perception of emotions and feelings. This shift involves a profound reorganization of personal identity, roles, and relational dynamics, as the family member gradually comes to accept and embrace the responsibilities inherent in caregiving. Rather than being a static or solely reactive experience, this transition reflects an active and ongoing process of meaning-making and role negotiation, where the individual redefines their sense of self in relation to the care recipient and their new circumstances.

Recent studies question the view of caregiving as only stressful. Han (2023) [34] found that caregiving transitions do not always increase depressive symptoms; sometimes, caregiving even lessens the negative effects of the care recipient’s disability on the caregiver’s mental health. This indicates that embracing the caregiving role within one’s identity can promote positive adjustment. Social support and resilience are also key in managing caregiving stress [34]. Geschke et al. (2024) [35] showed that higher resilience was linked to lower caregiver burden during COVID-19 lockdowns, highlighting how personal and social resources aid adaptation. Building resilience often means accepting and finding meaning in the caregiving role [35]. Finally, the narrative identity framework explains how caregivers reshape their self-stories to handle new challenges. Mroz et al. (2024) argue this improves stress models by showing how families move from shock to acceptance and active caregiving [36].

At the heart of this grounded theory lies the core category “A Silent and Certain Willingness to Care”. This concept captures participants’ portrayal of caregiving not merely as a responsibility but as a deeply meaningful, almost sacred act of love and moral duty. The decision to care, often not consciously chosen but naturally embraced, reflects a profound relational commitment. Many described their caregiving journey as a “calling,” driven by strong emotional bonds and a readiness to endure physical and psychological challenges to protect the dignity and identity of their loved one. This view resonates with recent research challenging the traditional perception of caregiving solely as a source of stress. For example, Kropf and Schmidhuber (2024) emphasize the ethical importance of moral identity in family caregivers, showing that caregiving reinforces and expresses their moral self, which is crucial for delivering compassionate care [37]. The idea of “beauty” in sacrifice illustrates how caregivers often find personal growth, emotional closeness, and spiritual fulfillment in their role despite—or perhaps through—their suffering. Supporting this, Uzun et al. (2024) highlight in a scoping review the spiritual needs of family caregivers in palliative care, underscoring that addressing existential concerns and fostering meaning-making can ease caregiver burden [38]. Together, these insights position caregiving as an act of existential and continuous becoming, reinforcing that the caregiving experience is best understood not as a static condition but as a dynamic, evolving process that redefines the caregiver’s sense of self, relationships, and life meaning over time.

The core category is closely intertwined with the four theoretical categories that emerged during the analytic process, each illuminating a different aspect of the complex transition from family member to informal caregiver. The first category, “The Changing Daily Life: Sacrifice,” captures the emotional disorientation following the initial diagnosis of AD, triggering a profound sense of loss and anticipatory grief. This phase marks the onset of an existential shift, where the willingness to care is not yet fully conscious but begins to take root and evolve. This is consistent with Kokorelias et al. (2021), who observed that caregivers often face a period of uncertainty and emotional upheaval immediately after diagnosis, requiring support to navigate this difficult transition [39]. The second category, “Feeling Powerless,” describes the internal conflict caregivers endure as they struggle to reconcile affection, responsibility, and fear—dilemmas that gradually transform into moral commitment. Here, sacrifice emerges not as an obligation but as a voluntary act rooted in relational ethics. Herron et al. (2020) support this, highlighting how caregivers confront emotional burdens while adapting to the changing needs of those with dementia, underscoring the moral complexities of caregiving decisions [40]. The third category, “Losing Oneself”, illustrates the tangible adjustments caregivers make in daily routines, priorities, and identities to meet caregiving demands. This pragmatic reorganization reflects caregivers’ growing emotional investment and deliberate choice to prioritize their loved one’s needs, embodying the “beauty of sacrifice” in concrete ways. Kokorelias et al. (2021) also emphasize that caregivers often undergo profound lifestyle changes, reshaping both personal and professional spheres to accommodate caregiving [39].

Finally, the fourth category, “Sharing with Others: Seeking Balance in Care”, signifies the full integration of the caregiving role. At this stage, caregivers not only provide physical support but also become protectors of their loved one’s dignity, identity, and humanity. This represents the culmination of caregiving as a moral journey—an enduring act of love driven by a deep, willing commitment despite hardships. Mroz et al. (2023) similarly note that caregivers develop a strong sense of responsibility and identity tied to their role, shaping their readiness for future caregiving challenges [41]. Together, these categories trace a trajectory characterized not just by burden and stress but by emotional depth, moral agency, and profound meaning, offering a rich and nuanced understanding of the caregiving experience in advanced AD.

Importantly, our study highlights the transformative role of peer support and psychoeducational interventions in the lives of informal caregivers, resonating with prior evidence that such interventions can reduce caregiver burden and promote well-being [42]. Recent research has shown that peer-led schemes for dementia caregivers not only enhance emotional well-being but also reduce social isolation by fostering a sense of community and shared understanding [43]. Similarly, digital peer-to-peer support programmes have demonstrated promising results in delivering scalable, accessible support to caregivers of patients with chronic conditions [44].

Additionally, combined psychoeducational and mindfulness interventions have generated positive perceived and physiological outcomes in caregivers, enhancing their coping skills and resilience [45].

Systematic reviews further confirm the effectiveness of online and videoconferencing support models, particularly for caregivers of people with dementia and other chronic diseases, underlining the potential of technology-mediated peer connections to mitigate stress and improve quality of life. These findings strongly align with our participants’ experiences, illustrating how connecting with peers who share similar caregiving trajectories can empower individuals to reframe their roles, reduce isolation, and foster mutual resilience. By integrating these interventions into routine caregiver support pathways, healthcare systems may better address the multifaceted needs of informal caregivers, promoting a more holistic and sustainable approach to caregiver well-being.

### 4.1. Study Limitations

While this study offers rich insights into the caregiving experience in advanced AD, it is not without limitations. The sample was composed of a small number of participants, primarily from a single cultural and healthcare context, which may limit the transferability of the findings to broader populations. Moreover, the retrospective nature of some accounts may have introduced recall bias, especially in narrating emotionally intense or prolonged experiences. Additionally, since participants were self-selected, it is possible that those who chose to take part in the study had particularly strong caregiving identities or motivations, potentially skewing the data towards more reflective or engaged narratives. It is important to acknowledge that detailed information on participants’ socioeconomic status, ethnicity, and use of domestic help was not systematically collected through the sociodemographic questionnaire. This limits the ability to fully contextualize how such factors may have influenced individual caregiving trajectories. Furthermore, the study did not specifically explore other concurrent caregiving responsibilities that participants might have had (e.g., caring for children or elderly relatives), which could have affected their coping capacity and overall caregiving experience. Future research should consider these additional caregiving roles to provide a more comprehensive understanding of the challenges faced by informal caregivers. Finally, although our analysis identified broad phases in the caregiving process, the study did not systematically explore in depth the specific transitional moments or shared events that might trigger movement from one phase to the next. This limits our understanding of the nuanced similarities and critical turning points across different caregiving trajectories.

### 4.2. Future Directions and Implications for Practice

Future research could benefit from including a more diverse sample across different cultural, socioeconomic, and healthcare settings to better understand the contextual factors shaping the caregiving transition. Longitudinal designs might also capture the evolution of the caregiving identity over time, offering further insight into how support needs and emotional responses change throughout the disease trajectory. From a clinical perspective, our findings suggest the need to move beyond a deficit-based model of caregiving support and toward one that acknowledges caregivers’ emotional and existential investments. Psychoeducational interventions, counseling, and peer support groups should be tailored not only to reduce burden but also to reinforce meaning, moral agency, and resilience. In line with approaches developed in other conditions—such as family-based skills training for carers of people with eating disorders—future programs could integrate practical tools to strengthen caregivers’ coping strategies and sense of efficacy. In the Italian context, where structured caregiver-focused interventions are still limited, our findings highlight the importance of developing policies and services that actively support family carers as co-participants in care, inspired by best practices from other healthcare fields. Healthcare professionals must be trained to recognize the relational and ethical dimensions of caregiving and to engage families as active, valued partners in the care process. Future research could benefit from longitudinal designs or more detailed narrative mapping to capture the dynamics of these transitions and to identify common milestones that shape the evolution of the caregiving experience.

## 5. Conclusions

This study provides a grounded understanding of how family members become informal caregivers for individuals with advanced AD. It highlights caregiving as a deeply human, morally infused process, where love, sacrifice, and identity intertwine. By centering on the core category of “The Willingness to Care and the Beauty of Sacrifice,” we have illuminated an often-overlooked dimension of the caregiving experience—one marked not only by burden but also by meaning, commitment, and emotional depth.

Importantly, our findings emphasize the diversity of caregiving arrangements—including both co-residing and non-cohabiting family caregivers—and the multifaceted nature of becoming an informal caregiver for a loved one with advanced AD. These insights call for a reframing of caregiving policies and practices that fully embrace the lived realities of caregivers and promote more empathetic, responsive, and flexible models of care that acknowledge this complexity.

## Data Availability

Data is contained within the article.

## Public Involvement Statement

Caregivers were involved in this study as participants through individual semi-structured interviews. Their experiential knowledge was central to the generation of data and the development of categories. However, they were not involved in the design of the study, development of the interview guide, data analysis, or dissemination of the findings.

## Guidelines and Standards Statement

This manuscript was drafted in accordance with the Consolidated Criteria for Reporting Qualitative Research (COREQ) checklist [26] for qualitative research based on the Grounded Theory methodology.

## Appendix A. Semi-Structured Interview Guide

1. Opening and Contextualization

- “Can you tell me about your experience of becoming a caregiver for your loved one with Alzheimer’s disease?”

2. The Beginning of the Caregiving Journey

- “When and how did you first realize that your loved one needed your help or care?”
- “What were the first changes you noticed in their behavior or abilities?”
- “Can you describe the moment you understood you were becoming their caregiver?”

3. Relational Transformation

- “How has your relationship with your loved one changed since you took on the caregiver role?”
- “Do you feel your emotional bond has evolved? In what ways?”
- “What aspects of being a daughter/son/spouse/sibling are still present, and what has changed?”

4. Role Assumption and Caregiver Identity

- “How would you describe the process of becoming a caregiver?”
- “Did you ever feel unprepared or unsure about this role?”
- “How do you define yourself today in relation to your loved one?”

5. Daily Life and Practical Dimension

- “What are the main tasks and responsibilities you manage on a daily basis?”
- “How did you learn to handle these tasks?”
- “Can you describe a typical day for you as a caregiver?”

6. Personal and Emotional Impact

- “How has caregiving affected your emotional well-being?”
- “Have there been moments of crisis, uncertainty, or personal growth?”
- “What has helped you cope during difficult times?”

7. Resources, Support, and Gaps

- “Have you received any form of support—family, professional, or social?”
- “What kind of help or resources do you think you would need?”
- “Is there something you wish others (professionals, friends, institutions) better understood about your experience?”

8. Reflection and Meaning-Making

- “Looking back, how do you make sense of your caregiving journey?”
- “What have you learned about yourself throughout this experience?”
- “If you had to describe this experience in a few words, what would they be?”

9. Closing

- “Is there anything else you’d like to share that we haven’t talked about?”
- “How did it feel to talk about your experience today?”
